# EEG alpha/delta ratio at different propofol-induced sedation levels in children during procedural sedation

**DOI:** 10.1186/s12871-026-03667-5

**Published:** 2026-02-09

**Authors:** Rayan Hojeij, Eva Tschiedel, Anna Daniels, Carolina A. Joist, Constantin M. Joist, Pia Brensing, Luisa Paul, Sandra Greve, Ursula Felderhoff-Müser, Christian Dohna-Schwake, Nora Bruns

**Affiliations:** 1 Department of Pediatrics I, Neonatology, Pediatric Intensive Care Medicine, Pediatric Neurology, and Pediatric Infectious Diseases, University Hospital Essen, University of Duisburg-Essen, Essen, Germany; 2 C-TNBS, Centre for Translational Neuro-and Behavioural Sciences, University Hospital Essen, University of Duisburg-Essen, Essen, Germany; 3 Department of Pediatric Cardiology/Congenital Cardiology, Heidelberg University Medical Center, Heidelberg, Germany

**Keywords:** Alpha/delta ratio, EEG, Frequency spectra, Pediatric, Procedural sedation, Propofol

## Abstract

**Introduction:**

The alpha-delta ratio (ADR) represents the relationship between alpha and delta waves and has emerged as a potential marker of hypnotic depth. However, the trajectory of ADR across pediatric age groups and hypnotic depths during propofol sedation has not been characterized. This study aimed to investigate ADR patterns after midazolam premedication plus propofol sedation across different age groups and hypnotic depths.

**Methods:**

This post-hoc analysis from a prospective observational study at a tertiary PICU (October 2020-December 2022) included children aged 6 months to 17.9 years undergoing procedural sedation with propofol. EEGs were recorded using BrainTrend 2.0 monitors with the electrodes placed at Fp1, Fp2, and FpZ positions. Hypnotic depth was assessed using the Comfort Scale (CS) every 5 minutes. The ADR was calculated as relative alpha power divided by relative delta power. Age-stratified analyses were performed (<3, 3-5, 6-11, 12-17 years).

**Results:**

Of 176 patients, 165 had eligible EEG recordings with 1,464 paired CS-EEG observations (8.9±3.5/patient). Median age was 8.8 years (IQR 3.9-14.0). Relative alpha power increased and delta power decreased with deepening hypnosis until CS=12, then reversed at deeper levels. Concordantly, the ADR increased with hypnotic depth, peaking around CS=12, then declined. The total power showed a U-shaped trajectory. These patterns were consistent across all age groups with no relevant age-related differences.

**Discussion:**

The ADR exhibited a characteristic non-linear trajectory at different depths of pediatric propofol-induced hypnosis, with reversals at very deep hypnosis levels. The curved ADR trajectory suggests that additional clinical assessment and propofol dosing information are essential for accurate interpretation of hypnotic depth.

**Supplementary Information:**

The online version contains supplementary material available at 10.1186/s12871-026-03667-5.

## Introduction

Electroencephalography (EEG) provides insights into cortical function and has emerged as an important tool for monitoring brain activity during sedation and anesthesia [1, 2]. Among the quantitative EEG (qEEG) measures that facilitate point-of-care interpretation by non-epileptologists, the alpha-delta ratio (ADR) has gained particular interest as a potential marker of hypnotic depth, impending emergence delirium (ED), and cerebral integrity in neurocritical care from pediatric to adult populations [3–10]. The ADR represents the relationship between higher-frequency alpha activity and lower-frequency delta waves and is associated with emergence patterns and neurophysiological changes induced by anesthetic agents which stimulate GABA and Alpha-adrenergic neurotransmission such as propofol, midazolam, and dexmedetomidine for procedural sedation [5, 6].

Propofol, a commonly used intravenous anesthetic for procedural sedation in children, exerts dose-dependent effects on EEG patterns [11]. However, the trajectory of ADR across different pediatric age groups and hypnotic depths has not yet been characterized during propofol-induced hypnosis. Prior research suggests that age-related variations of neurophysiological responses to anesthesia may influence EEG patterns [12, 13] and that ADR varies across pediatric ages and brain regions [14]. Understanding physiological variations and putting them into context with deviations thereof is essential for optimizing EEG-guided sedation or anesthesia protocols, minimizing adverse outcomes, and improving patient safety during procedural sedation.

This study aims to investigate the course of ADR during midazolam plus propofol sedation across different age groups and hypnotic depths during procedural sedation with preserved spontaneous breathing. This study is a *post-hoc* analysis of a prospective observational study on the correlation of the Narcotrend index with clinical hypnotic depth assessment during procedural sedation with propofol in children [15].

## Methods

The here-presented data is a *post-hoc* analysis of a previously published prospective study on the correlation of the Narcotrend index with the comfort score during procedural sedation [15]. that has been re-analyzed with respect to aEEG changes [16]. Children between 6 months and 17.9 years of age undergoing procedural sedation with propofol after midazolam premedication in a tertiary PICU of the University Hospital Essen were prospectively included between October 2020 and December 2022. Reasons for exclusion were underlying neurological diseases potentially impairing Comfort scale (CS) scoring, known EEG abnormalities, prior participation in this study, and anticipated use of ketamine or remifentanil during the procedure. Patients who unexpectedly received ketamine or remifentanil during the sedation were retrospectively excluded.

Eligible procedures were endoscopies, bronchoscopies, biopsies, and punctures including drain placements. Shortly after the initiation of the study, routine sedation regimes for muscle biopsies and bronchoscopies were changed to remifentanil + propofol, resulting in secondary exclusion of these procedures.

### Sedation

Sedation was performed by experienced pediatric intensivists (A.D., C.D.S., E.T.) in the PICU following international guidelines [17, 18]. According to our standard procedural sedation regime, intravenous (i.v.) midazolam (0.05 mg/kg, maximum 2 mg) was administered as premedication before placement of the EEG electrodes (section EEG recording). Within 2 to 3 min of administration, Midazolam was followed by an i.v. propofol induction bolus (1 mg/kg) and continuous propofol infusion (10 mg/kg/h). The level of hypnotic depth was optimized by clinical assessment (CS target range 11–14) via titration of propofol boli (1 mg/kg) or adjustment of the continuous infusion rate as required according to the clinical setting. Propofol administration was immediately stopped at the end of the procedure. All patients received prophylactic oxygen via a nasal cannula throughout the sedation.

CS scores < 11 were considered too deep hypnosis and > 14 as light hypnosis.

### Clinical measurement of hypnotic depth

Hypnotic depth was assessed using the CS [19], the department’s standard tool for evaluating hypnotic depth during procedural sedation. Assessments were performed by one of two trained medical students (CAJ or CMJ), who were not involved in the sedation or procedure and were present solely for study purposes. Given that the CS has demonstrated substantial to excellent inter-rater reliability in prior studies [20, 21], we did not perform a separate reliability assessment. Objectivity of CS scoring was further supported through the use of standardized scoring sheets and concurrent documentation of vital signs (Supplementary Tables 1 and 2).

The CS was recorded every five minutes from the beginning of the sedation, along with additional responses to intervention-related stimuli. After the procedure, a standardized painful stimulus was applied to the sternum every five minutes until eye-opening. Like during the procedure, the reaction to the stimulus was recorded as part of the CS assessment.

### Clinical documentation

The CS and propofol infusion rate were documented manually in the case report form (CRF) at the beginning of sedation and every five minutes until eye-opening by a medical doctorate student uninvolved in the sedation or the procedure. Propofol bolus application and adverse events were documented whenever they occurred. Documentation from the CRFs was transferred to Excel, including pseudonyms and time stamps.

### EEG recording

**Fig. 1:**
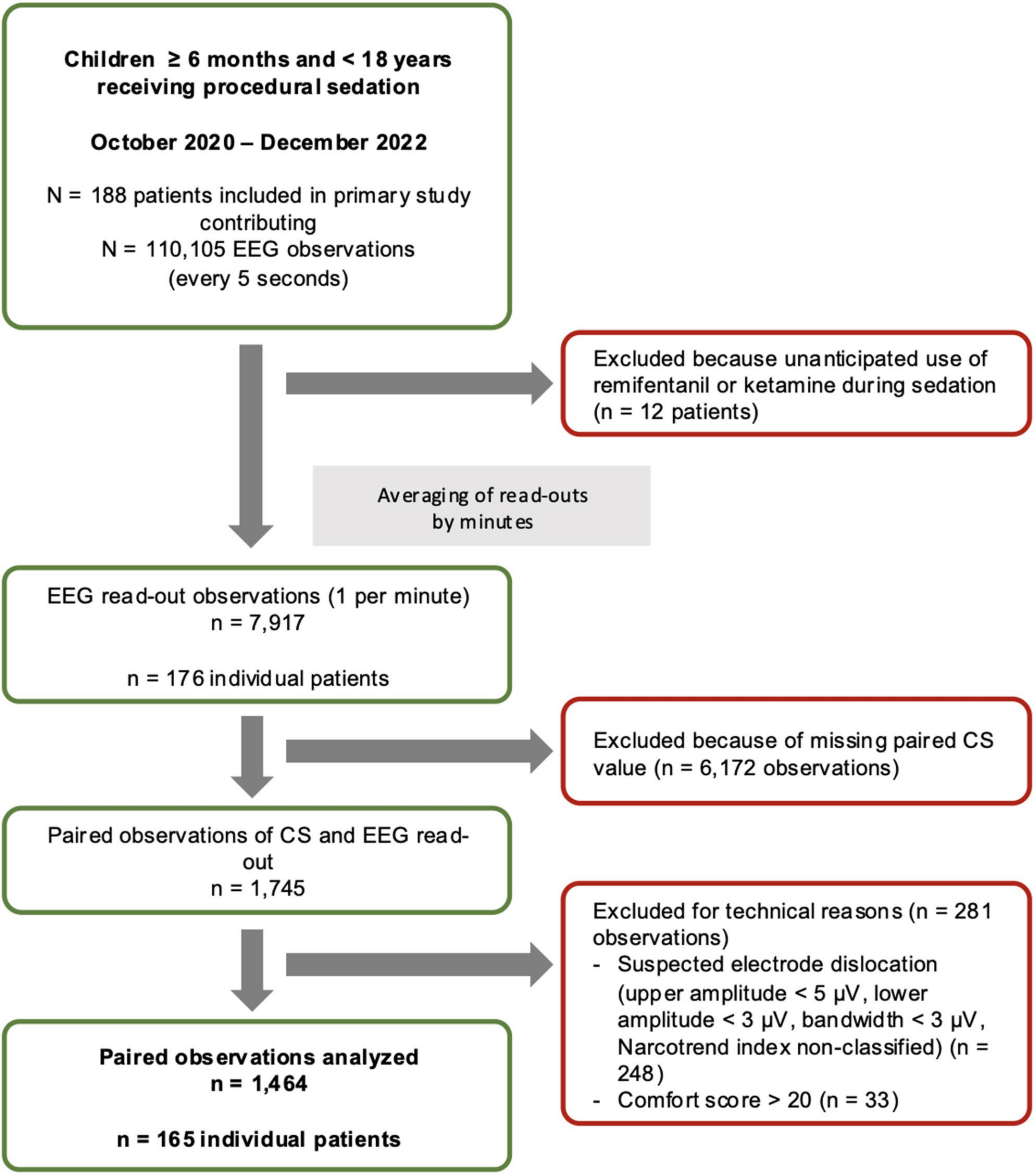
Patient flow and data processing

EEGs recordings were obtained using BrainTrend 2.0 monitors (MT Monitortechnik, Bad Bramsted, Germany). While the patient was still awake, three hydrogel electrodes were placed on the forehead in the Fp1, Fp2, and FpZ position according to the international 10–20 system (Fig. 1). Skin preparation was performed with OneStep EEG Gel Abrasiv plus® (H + H Medizinprodukte, Münster, Germany) until impedance values were < 10 kΩ. EEGs were discontinued after awakening.

Because the recorded EEGs were not part of the clinical routine, all EEGs were recorded pseudonymized without patient-identifying information contained in the EEG file. Vital sign monitoring was carried out according to the clinical routine.

### EEG processing (manufacturer)

EEGs were processed by the manufacturer of the BrainTrend monitor for the purpose of the post-hoc analysis.

The total power (µv^2^) was calculated for predefined frequency ranges as described in the manufacturer’s manual (delta: 0.5–3.5 Hz, theta: 3.5–7.5 Hz, alpha: 7.5–12.5 Hz, beta: > 12.5 Hz) for each five-second epoch. The relative power was calculated as proportion of the total power. The read-outs received from the manufacturer contained the pseudonym, time stamp, total power, relative band power, and information on automated artifact detection and electromyogram (EMG) intensity.

### EEG cleaning and merging of automated and manual data (study site)

The EEG read-outs were cleaned by removing observations with artifacts and muscle activity (identified by automated artifact detection and observations with EMG index > 20 of 100) and suspected electrode dislocation (upper amplitude measurements < 5 µV, lower amplitude < 3 µV, bandwidth < 5 µV, Narcotrend index non-classifiable). The measurements of the cleaned EEG read-outs were averaged by minute and matched by time stamp (at minute level) and pseudonym with the manual records. Because there were more observations per EEG read-out than by manual recording (1 min-averaged values versus 5 min), the number of paired observations was determined by the number of manual observations per patient. To avoid distortion of results from muscle artifacts in partially or fully awake patients, further analyses included only paired observations with a CS ≤ 20.

### Statistical analyses

Continuous variables are presented as mean if evenly distributed and as median if skewed. Discrete variables are summarized as counts and percentages. Age-stratified analyses were conducted by age groups (< 3 years, 3–5 years. 6–11 years, 12–17 years) to maintain the same structure as in the previous publications from this dataset [15, 16].

The alpha/delta ratio was calculated by dividing the relative alpha power by the relative delta power. For relative alpha and delta power, the ADR, and the total power, we calculated median and interquartile ranges for each age group and hypnotic depth category (too deep, target depth, light). For more in-depth visualization of frequency trajectories depending on the CS, we calculated moving median and IQR values for each CS value, which included the measurement from the CS value of interest along with the measurements from the neighboring CS values (e.g., for CS 13, all measurement from CS 12 through CS 14 were included). We chose this approach to account for suspected slight imprecisions during CS assessments that may occur due to the very fine scaling of the CS and to produce smoother curves for interpretation.

All statistical analyses were performed using SAS Enterprise Guide Version 8.3 (SAS Institute Inc., Cary, NC, USA). Figures were produced using SAS Enterprise Guide and Microsoft Office PowerPoint.

### Ethics approval

The study was approved by the local ethics committee (19–8728-BO, 21–10306-BO). Written informed consent was obtained from the legal guardians of all included patients. Clinical trial number: not applicable.

## Results

Of 176 patients included in the original study, 165 had eligible EEG recordings after completion of the data cleaning procedure (Fig. 1). In sum, 1464 paired observations consisting of CS and EEG read-outs were analyzed (8.9 ± 3.5/per patient). The median patient age was 8.8 years (IQR 3.9–14.0, *n* = 5 < 12 months) and the median weight was 28.0 kg (IQR 17.0–50.0) (Table 1). Procedures were most frequently biopsies (liver, kidney, skin, muscle, thyroid; *n* = 72 (43.7%)), punctures (lumbal, pleural drainage, ascites drainage, bone marrow, joint; *n* = 48 (29.1%)), and esophagogastroduodenoscopies/transesophageal echocardiographies (*n* = 45 (27.3%)) with a median duration of 19 min.

**Table 1:** Clinical and periprocedural information

	Overall	0–2 years	3–5 years	6–11 years	12–17 years
*N (%)*	165 (100%)	16 (9.7%)	45 (27.3%)	43 (26.0%)	61 (37.0%)
Procedure*					
Esophagogastroduodenoscopy, placement of percutaneous gastroenterostomy, transesophageal echocardiography; *n (%)*	45 (27.3%)	4 (2.4%)	14 (8.48%)	13 (7.88%)	14 (8.48%)
Colonoscopy, rectoscopy; *n (%)*	5 (3.0%)	0 (0.0%)	2 (1.21%)	2 (1.21%)	1 (0.61%)
Placement of pH-metry probe; *n (%)*	3 (1.8%)	1 (0.61%)	0 (0.00%)	2 (1.21%)	0 (0.00%)
Bronchoscopy; *n (%)*	6 (3.6%)	4 (2.42%)	0 (0.00%)	2 (1.21%)	0 (0.00%)
Biopsy (liver, kidney, skin, muscle, thyroid); *n (%)*	72 (43.6%)	4 (2.42%)	16 (9.70%)	15 (9.09%)	37 (22.42%)
Puncture (lumbal, pleural drainage, ascites drainage, bone marrow, joint); *n (%)*	48 (29.1%)	5 (3.03%)	13 (7.88%)	14 (8.48%)	16 (9.70%)
Catheter placement or removal (central venous catheter, Shaldon, Broviac); *n (%)*	14 (8.5%)	1 (0.61%)	4 (2.42%)	4 (2.42%)	5 (3.03%)
Multiple procedures; *n (%)*	21 (12.7%)	3 (18.8%)	2 (4.4%)	7 (16.3%)	9 (14.8%)
Duration of propofol administration [min], *median (IQR)*	19.0 (14.0–25.0)	24 (20.0–30.0)	16 (14.0–20.0)	17 (12.0–28.0)	20 (14.0–30.0)
Time until eye-opening [min], *median (IQR)*	16.0 (10.0–30.0)	26.5 (21–33.5.5)	20.0 (14.0–25.0)	17.0 (10.0–23.0)	13.0 (7.0–16.0)
Propofol dose					
Total dose [mg/kg], *median (IQR)*	17.0 (12.9–23.2)	19.0 (14.9–23)	22.4 (15.7–27.3)	19.1 (14.0 −23.3)	12.9 (10.2–17.1)
Induction dose via bolus application [mg/kg], *median (IQR)*	2.5 (1.8–4.1)	3.9 (2.4–4.3)	2.4 (1.9–3.6)	2.6 (1.7–4.1)	2.4 (1.7–3.6)
Maintenance dose via syringe pump ± intermittent bolus application [mg/kg], *median (IQR)*	5.6 (4.1–7.7)	7.4 (6.0 −8.9)	6.3 (5.2–7.9)	6.0 (4.4–7.2)	4.3 (3.1–5.9)

*IQR* Interquartile range

*Multiple procedures within the same sedation possible

**Fig. 2:**
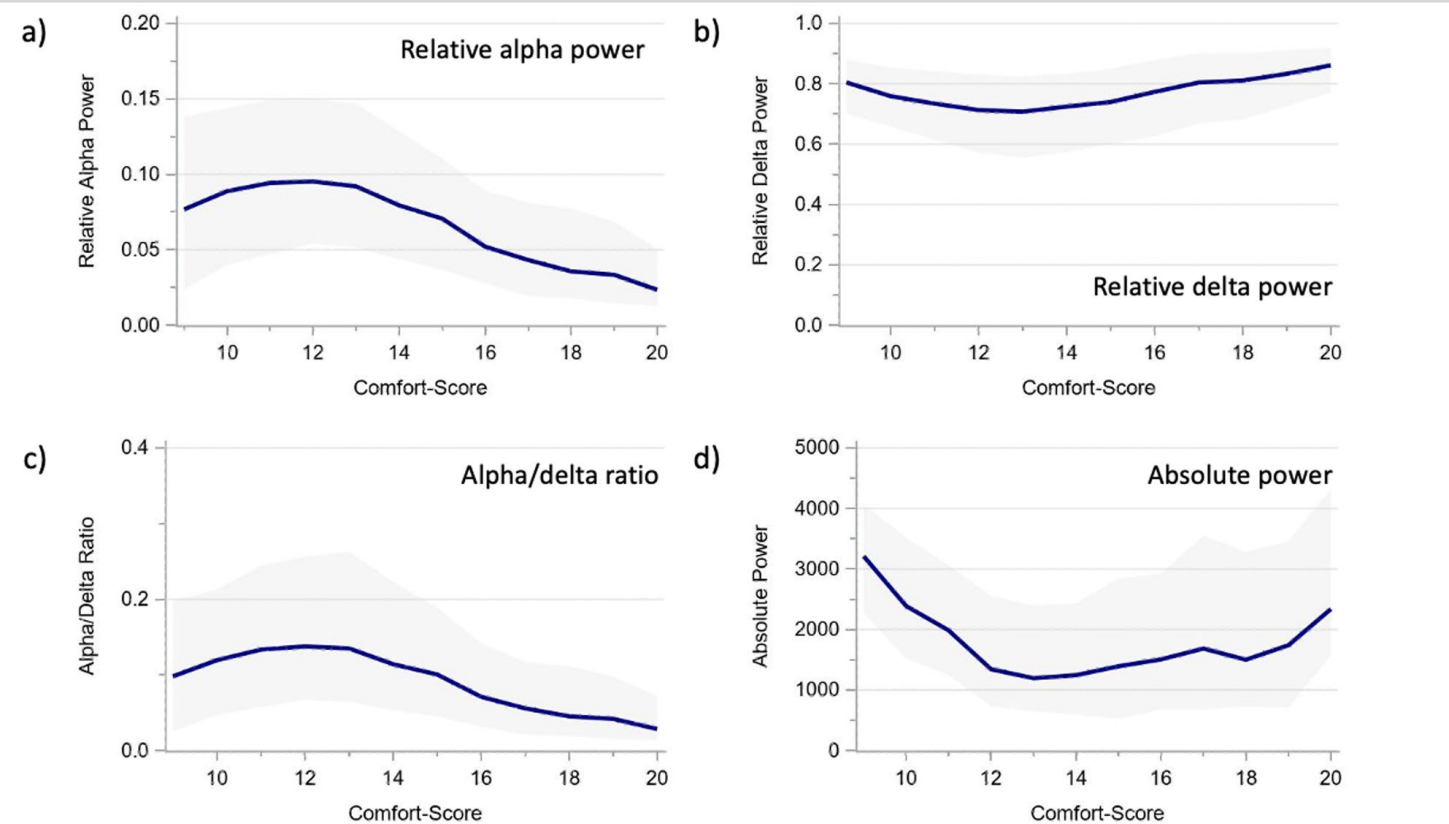
Relative alpha and delta power, alpha/delta ratio, and total power of the overall cohort across comfort scale values. **a** Relative alpha power. **b** Relative delta power. **c** Alpha/delta ratio. **d** Total power The presented values represent moving medians (blue line) and moving interquartile ranges (grey band)

The relative alpha power increased at deeper hypnosis, peaking around CS = 12 and then declined. Conversely, the relative delta power decreased until CS = 12 and then increased at deeper levels (Table 2; Fig. 2). The ADR increased with deepening hypnosis. During deep and too deep hypnosis (CS ≤ 12), all three measurements tended to reverse. The power did not evolve linearly but presented a U-shape that also showed higher values below CS 13 (Table 2; Fig. 2).

**Table 2:** Relative frequency power, alpha/delta ratio, and total power by sedation depth and age

	Sedation depth*	Overall	0–2 years	3–5 years	6–11 years	12–17 years
Paired observations, *n*	-	1464	148	401	365	551
Relative alpha power (%), *median (IQR)*	Light	4 (2–8)	5 (4–6)	4 (2–9)	4 (1–7)	3 (1–7)
	Target depth	9 (5–14)	9 (5–17)	10 (6–16)	8 (5–12)	8 (4–13)
	Too deep	10 (6–15)	8 (7–13)	15 (7–17)	9 (7–11)	9 (4–14)
Relative delta power (%), *median (IQR)*	Light	82 (69–91)	77 (64–82)	81 (66–90)	82 (71–93)	84 (69–92)
	Target depth	72 (56–83)	67 (51–76)	67 (49–79)	72 (60–84)	75 (62–87)
	Too deep	72 (62–83)	70 (59–74)	66 (51–81)	75 (70–79)	75 (64–89)
Alpha/delta ratio (%), *median (IQR)*	Light	5 (2–11)	8 (4–9)	5 (2–14)	4 (1–9)	4 (2–11)
	Target depth	12 (6–25)	13 (7–32)	17 (7–33)	11 (5–20)	10 (5–22)
	Too deep	14 (7–23)	13 (10–18)	23 (9–32)	12 (8–16)	12 (4–22)
Total Power (µV^2^), *median (IQR)*	Light	1,807 (677–3,577)	1,082 (492–1,883)	2,173 (713–3,492)	2,039 (1,028 − 3,245)	1,546 (537–3,709)
	Target depth	1,208 (613–2,329)	798 (530–1,243)	1,166 (596–2,114)	1,538 (676–2,776)	1,283 (668–2,609)
	Too deep	1,583 (771–2,967)	1,074 (606–1,316)	1,593 (830–2,575)	2,444 (1,839–3,374)	1,365 (736–3,348)

*According to Comfort score target ranges specified in methods section

**Fig. 3:**
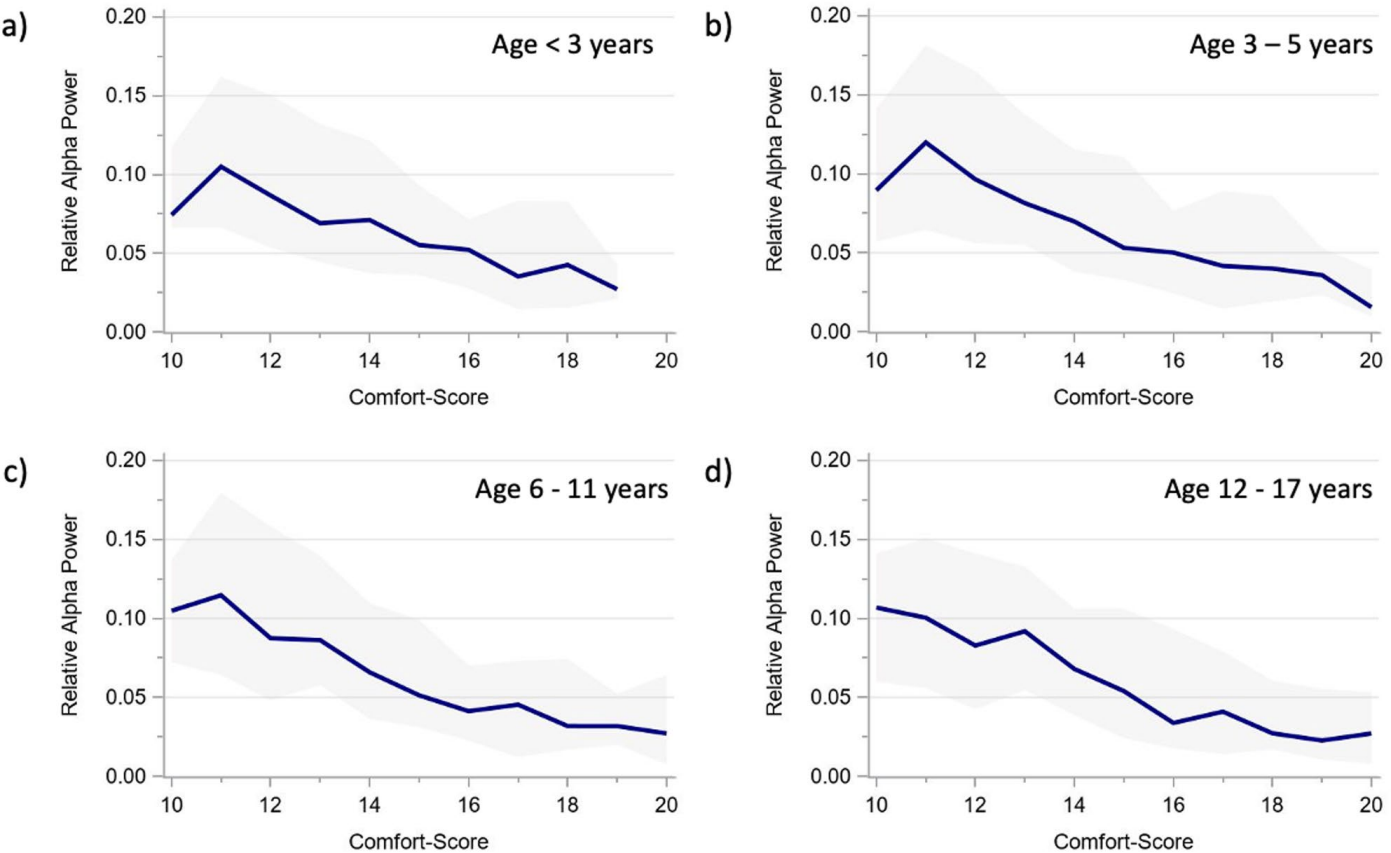
Relative alpha power across comfort scale values by age groups (**a**) < 3 years. **b** 3–5 years. **c** 6–11 years. **d** 12–17 years The presented values represent moving medians (blue line) and moving interquartile ranges (grey band)

**Fig. 4:**
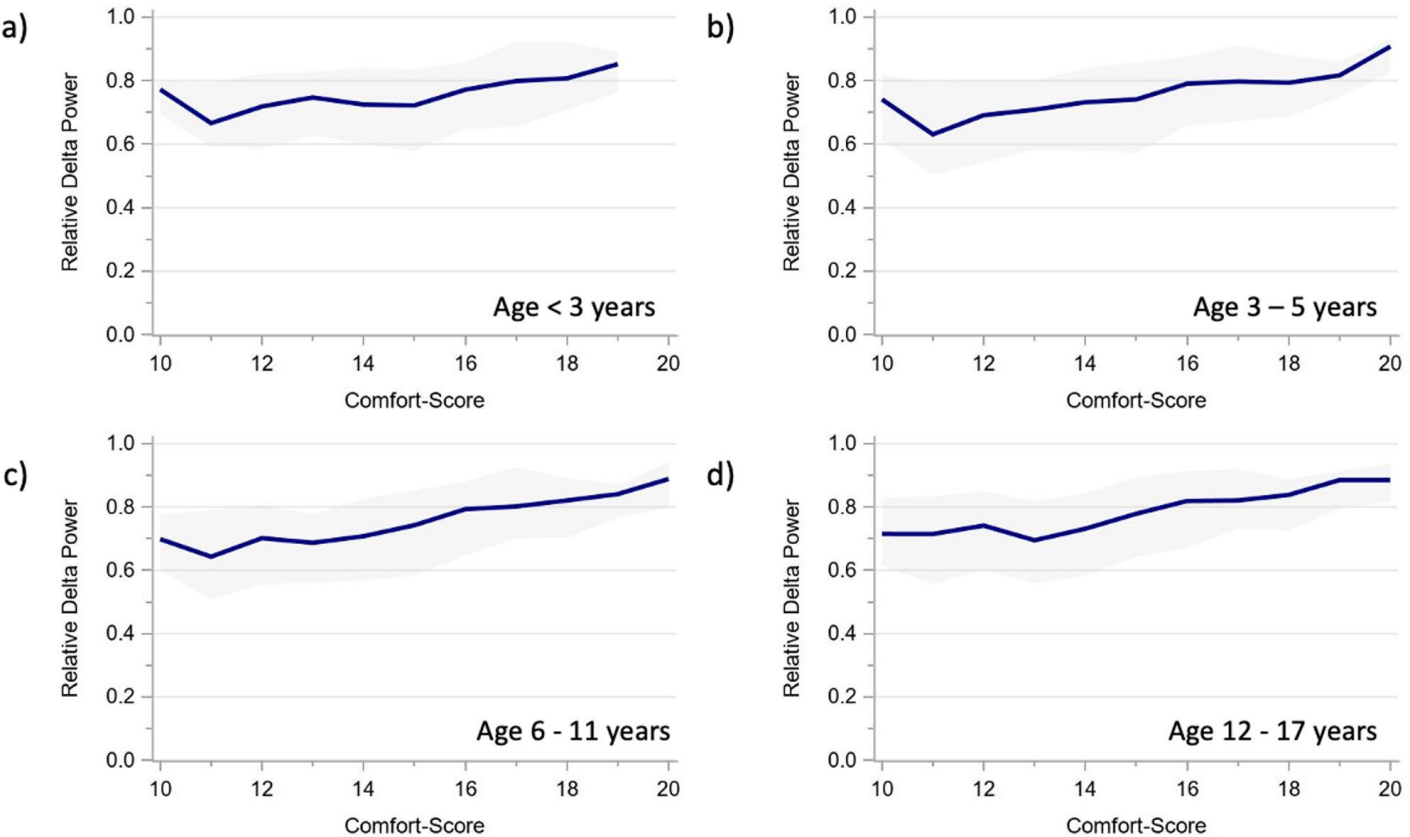
Relative delta power across comfort scale values by age groups (**a**) < 3 years. **b** 3–5 years. **c** 6–11 years. **d** 12–17 years The presented values represent moving medians (blue line) and moving interquartile ranges (grey band)

**Fig. 5:**
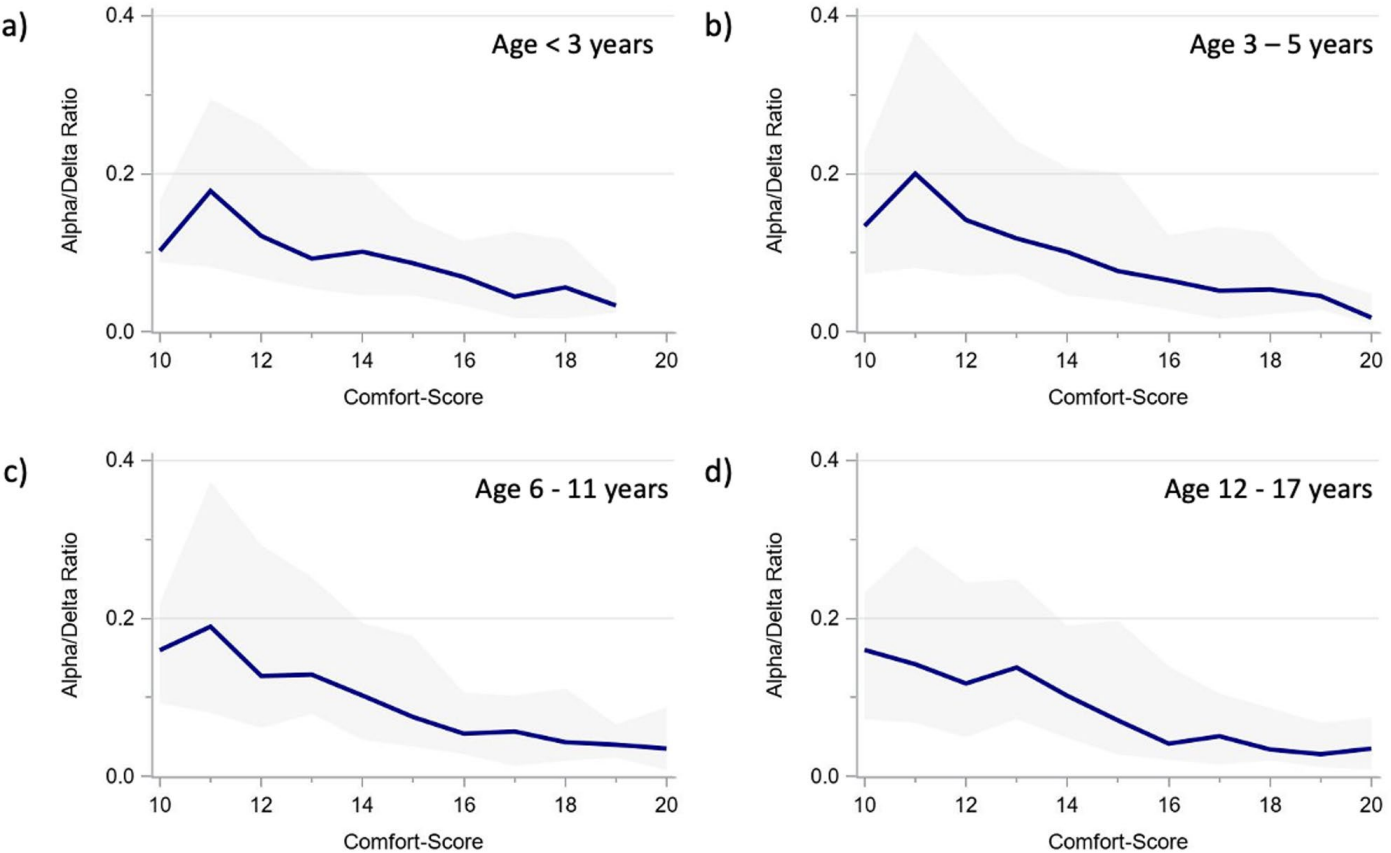
Alpha/delta ratio across comfort scale values by age groups (**a**) < 3 years. **b** 3–5 years. **c** 6–11 years. **d** 12–17 years. The presented values represent moving medians (blue line) and moving interquartile ranges (grey band)

**Fig. 6:**
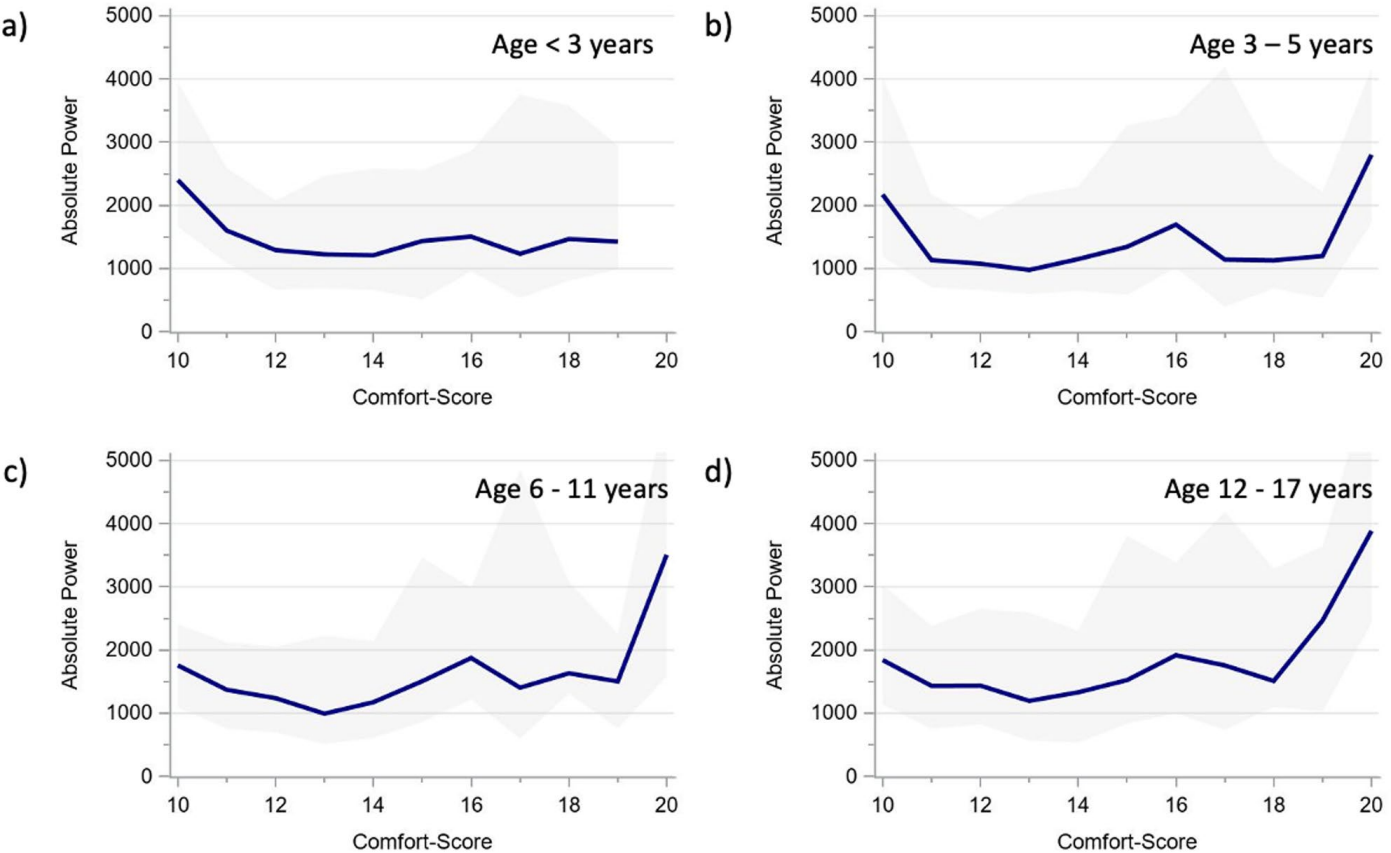
Total power across comfort scale values by age groups (**a**) < 3 years. **b** 3–5 years. **c** 6–11 years. **d** 12–17 years. The presented values represent moving medians (blue line) and moving interquartile ranges (grey band)

**Fig. 7:**
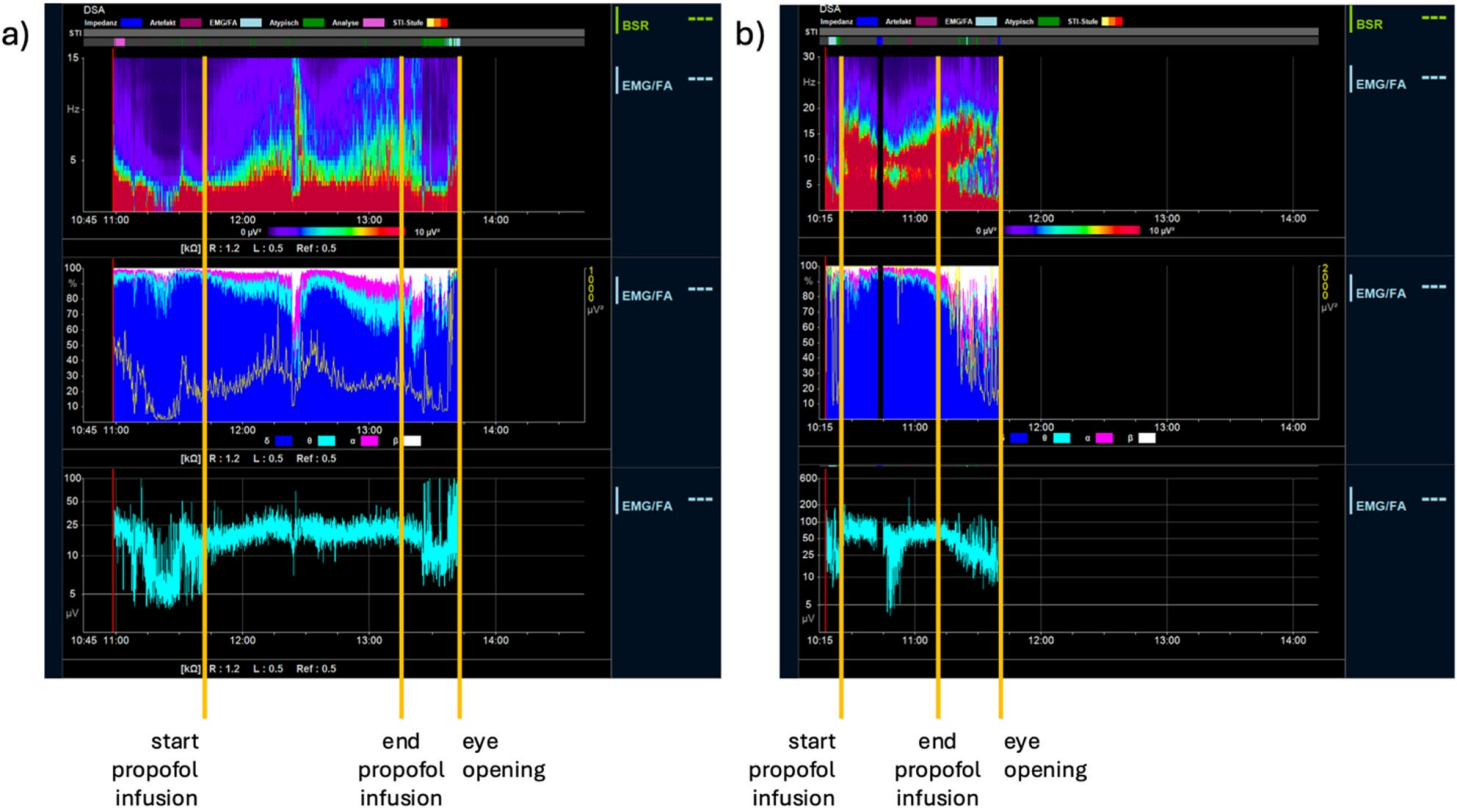
Example tracings of Density Spectral Array (upper), relative band power (intermediate), and amplitude-integrated EEG (lower) during propofol-induced procedural sedation in an infant aged 8 months (**a**) and a child aged 4 years **b**

The alpha and delta power showed very similar age-specific trajectories across CS scores (Figs. 3 and 4), as did the derived ADR (Fig. 5). The total power trajectories showed slightly more variation between the age groups but showed no clearly deviating trends (Fig. 6). The data underlying Figs. 2, 3, 4, 5 and 6 are presented in Supplementary Table 3 (all ages) and 4 (by age group) and Fig. 7 presents two example trajectories of density spectral array, relative bandpower, and amplitude-integrated EEG.

## Discussion

This prospective observational investigated the relationship of age with relative alpha and delta power, alpha-delta ratio (ADR), and total power during deep procedural sedation with midazolam and propofol. The ADR increased with hypnotic depth, peaking around a CS of 12 and declining at deeper hypnosis stages. The underlying relative alpha and delta power exhibited hypnotic depth-dependent distributions with rising alpha power and declining delta power until a CS of around 12 and reversal at deeper hypnotic levels. Power followed a U-shaped trajectory rather than a linear progression. All findings were consistent across the examined pediatric age groups.

Prior research has shown that escalating propofol doses to the point of burst suppression reduces alpha power [22]. This aligns with our inverse U-shaped finding: after peaking during deep hypnosis, alpha power—and consequently the ADR—declined at deeper hypnotic levels. These observations are consistent with known EEG and GABAergic effects of propofol. Beta and alpha activity dominate during light hypnosis, alpha and delta during adequate hypnosis, and delta during deep hypnosis. As a result, the alpha/delta ratio is low in light hypnosis, increases at appropriate hypnotic depth, and decreases again in deep hypnosis [23]. Due to its susceptibility to changes in both the numerator and the denominator, the alpha/delta ratio alone can be misleading when assessing hypnotic depth. Accurate assessment requires inspection of the raw EEG and the density spectral array to identify beta activity and slower delta waves [23].

An additional aspect that warrants consideration is the pharmacodynamic interaction between midazolam premedication and subsequent propofol administration. Both agents potentiate GABA_A_ receptor–mediated inhibition and may exert synergistic effects on thalamo-cortical oscillatory activity [24, 25]. Midazolam premedication has been shown to modify frontal EEG signatures under propofol anesthesia, including alterations of alpha and delta power distributions [6]. In our cohort, all patients received standardized low-dose midazolam immediately before propofol sedation, and it is therefore plausible that the observed peak of the alpha/delta ratio around CS = 12 reflects combined drug effects rather than propofol alone. Such interaction may accentuate alpha activity at intermediate hypnotic depths and further contribute to the non-linear ADR trajectory observed. Accordingly, ADR patterns and thresholds should be interpreted in the context of combined sedative regimens, and extrapolation from propofol-only anesthesia studies should be made with caution.

In pediatric settings, these changes should additionally be considered in the context of developmental neurophysiology. Age-related differences during sevoflurane anesthesia in the first year of life - with alpha oscillations occurring at the age of 6 months and alpha coherence from 10 months onwards - have been attributed to maturation of the thalamo-cortical circuitry [13]. Increasing EEG power from infancy until 6 years of age has been reported for sevoflurane anesthesia [26]. However, age-related changes of the ADR during propofol administration for sedation or general anesthesia in pediatric populations have not been reported to our knowledge. We found no relevant changes of the studied parameters across age groups. This discrepancy from sevoflurane-based studies may be explained by different age cohorts, which comprised infants 0–3 years of age in the study by Cornelissen et al. [13] versus infants ≥ 6 months up to adolescents aged 17 years in our study. Beyond age-cohort differences, discrepant findings compared with sevoflurane studies may also reflect agent-specific neurophysiology, as propofol (predominantly GABA_A_-mediated) and sevoflurane (multitarget volatile anesthetic) can produce distinct oscillatory EEG signatures and dose-dependent alpha/delta dynamics [22, 26]. In addition, our data were obtained during procedural sedation with preserved spontaneous breathing rather than general anesthesia, which may further limit direct comparability with sevoflurane anesthesia cohorts. Finally, our findings align with observations from neurologically healthy awake children between 1 month and 17 years, where relative alpha and delta power, ADR, and total power remained largely consistent in Fp1-Fp2 channels across age groups, contrasting with marked changes that were observed in C3-P3 and C4-P4 channels [14].

It is important to acknowledge that the Fp1–Fp2 channels are particularly susceptible to artifacts from muscle activity and eye movements, and cannot fully reflect global cortical dynamics. In particular, Akeju et al. showed that developmental changes in EEG power and oscillatory structure during anesthesia are region-specific and more evident in posterior cortical areas, reflecting maturation of thalamo-cortical and cortico-cortical networks [26]. Accordingly, the absence of marked age-related differences in ADR observed in our study likely reflects the limited spatial coverage of a frontal-only montage rather than a true lack of developmental effects. Furthermore, frontal EEG recordings may not fully capture propofol-induced alterations in posterior cortical regions that contribute to hypnotic depth. While frontal montages are commonly used in clinical sedation monitoring due to their practicality, these factors limit the generalizability of our findings to other electrode locations.

Several further limitations of our study require acknowledgement: Because the youngest age group, in which brain maturation is most rapid and finer stratification would have been optimal, was too small for subanalyses, it was collapsed into a single category, thereby limiting the generalizability of age-specific findings within this developmental period. For CS ranges that were unintentionally low or high, the number of observations is relatively small, limiting the reliability of the results for CS values < 11 and > 14. The small number of electrodes that were used limit the generalizability of our findings to other electrode positions such as C-P. The Fp1-Fp2 channel is highly susceptible to muscle artifacts in awake patients, which we addressed by excluding measurements with CS scores indicating agitation and with muscle artifacts indicated by the software. Furthermore, our measurements encompassed a minimal hypnotic depth of CS = 9. This limits the comparability with literature, which is predominantly on general anesthesia rather than deep hypnosis. No structured emergence delirium assessment was carried out due to the purpose of the main study, making it impossible to address this important aspect in this analysis, which would have been of particular interest, as a reduced ADR may be related to the occurrence of emergence delirium, in children, adults, and elderly patients [3, 4, 27]. Finally, because the analysis was descriptive and did not use mixed-effects models to explicitly partition within- and between-subject variability, future confirmatory studies should apply pre-specified hierarchical modeling approaches when testing sedation-depth or age effects.

Nonetheless, our study findings demonstrate that during procedural propofol sedation in children, frontal EEG exhibits quantifiable changes that correlate with the hypnotic depth. The curved ADR trajectory indicates that consideration of the administered propofol dose and boluses, together with clinical assessment, other quantitative EEG markers or raw EEG inspection is needed to distinguish between deepening and lightening hypnosis. Taken together, the observed trajectories of relative alpha and delta power, the derived ADR, and total power across hypnotic depths were coherent and physiologically plausible, reflecting the electrocortical response to propofol-induced hypnosis. Further research may identify ADR target ranges for propofol-induced procedural sedation to facilitate titration of hypnosis depth. In the critically ill children, the ADR should be additionally explored regarding its potential to identify patients at risk for delirium, as recently suggested [28].

## Conclusion

The ADR shows a characteristic trajectory during pediatric procedural propofol sedation with a peak at CS 12 and decline at deeper hypnosis levels. No relevant differences were observed between age-groups. While ADR monitoring may aid hypnosis guidance, information on the administered propofol dose and clinical markers remain essential for accurate interpretation.

## Supplementary Information

Supplementary Material 1.

Supplementary Material 2.

Supplementary Material 3.

## Data Availability

The datasets generated will be made available to any qualified researcher upon reasonable request.

## Abbreviations

ADR: Alpha/delta ratio
CRF: Case report form
CS: Comfort scale
ED: Emergence delirium
EEG: Electroencephalography
EMG: Electromyogram
IQR: Interquartile range
PICU: Pediatric intensive care unit
qEEG: Quantitative electroencephalography
