# Supplementary material for: EEG alpha/delta ratio at different propofol-induced sedation levels in children during procedural sedation
Source: BMC Anesthesiol. 2026 Feb 9;26:140. doi: 10.1186/s12871-026-03667-5 (PMC12931051; doi:10.1186/s12871-026-03667-5)
Supplement: Supplementary file 1 — Supplementary Material 1. [file 12871_2026_3667_MOESM1_ESM.docx]

**Supplementary Table 1:** Comfort Scale (total score ranging from 8 [deep sedation] to 40 [alert and agitated])

|  | **1** | **2** | **3** | **4** | **5** |
| --- | --- | --- | --- | --- | --- |
| **Alertness Lightly** | Deeply asleep | Asleep | Drowsy | Fully awake and alert | Hyper-alert |
| **Calmness or agitation** | Calm | Slightly anxious | Anxious | Very anxious | Panicky |
| **Crying** | Quiet breathing, no crying sounds | Occasional sobbing or moaning | Whining (monotone) | Crying | Screaming or shrieking |
| **Physical movement** | No movement | Occasional, slight movement | Slight movement often | Vigorous movement limited to extremities | Vigorous movement including torso and head |
| **Blood Pressure** | Less than baseline | Consistently at baseline | Occasional increase of 15% or more (1-3 episodes during observation period) | Multiple increases of 15% or more (>3 episodes) | Sustained increase >15% |
| **Heart rate** | Less than baseline | Consistently at baseline | Occasional increase of 15% or more (1-3 episodes during observation period) | Multiple increases of 15% or more (>3 episodes) | Sustained increase >15% |
| **Muscle Tone** | Muscles totally relaxed; no muscle tone | Reduced muscle tone | Normal muscle tone | Increased muscle tone and flexion of fingers and toes | Extreme muscle rigidity and flexion of fingers and toes |
| **Facial tension** | Facial muscles totally relaxed | Facial muscle tone normal; no facial muscle tension evident | Tension evident in some facial muscles | Tension evident throughout facial muscles | Facial muscles contorted and grimacing |

**Supplementary Table 2:** Blood Pressure and Heart rate reference range (values in parentheses +/- 15%)

| **Age** | **Diastolic blood pressure (mmHg)** | **Mean arterial blood pressure (mmHg)** | **Systolic blood pressure (mmHg)** | **HR (bpm)** |
| --- | --- | --- | --- | --- |
| **6 mo** | (37) 43-63 (73) | (43) 50-90 (104) | (68) 80-110 (127) | (94) 110-175 (201) |
| **1-3 y** | (39) 46-79 (91) | (43) 50-100 (115) | (68) 80-113 (130) | (68) 80-140 (161) |
| **4-6 y** | (40) 47-79 (91) | (47) 55-95 (110) | (68) 80-115 (132) | (64) 75-130 (150) |
| **7-10 y** | (44) 52-83 (96) | (51) 60-90 (104) | (71) 83-122 (140) | (64) 70-120 (138) |
| **11-13 y** | (49) 58-88 (101) | (55) 65-95 (110) | (81) 95-136 (156) | (51) 60-100 (115) |
| **14-16 y** | (47) 55-77 (89) | (60) 70-94 (108) | (85) 100-127 (146) | (42) 50-100 (115) |
